# Effects of alleles in crossbred pigs estimated for genomic prediction depend on their breed-of-origin

**DOI:** 10.1186/s12864-018-5126-7

**Published:** 2018-10-11

**Authors:** Claudia A Sevillano, Jan ten Napel, Simone E F Guimarães, Fabyano F Silva, Mario P L Calus

**Affiliations:** 1 Wageningen University & Research Animal Breeding and Genomics, P.O. Box 338, Wageningen, AH 6700 The Netherlands; 2 Topigs Norsvin Research Center, P.O. Box 43, Beuningen, 6640 AA The Netherlands; 3 Department of Animal Science, Universidade Federal de Viçosa, Viçosa, Minas 36570-000 Brazil

**Keywords:** Crossbred, Pig, Breed-of-origin, Genomic prediction

## Abstract

**Background:**

This study investigated if the allele effect of a given single nucleotide polymorphism (SNP) for crossbred performance in pigs estimated in a genomic prediction model differs depending on its breed-of-origin, and how these are related to estimated effects for purebred performance.

**Results:**

SNP-allele substitution effects were estimated for a commonly used SNP panel using a genomic best linear unbiased prediction model with breed-specific partial relationship matrices. Estimated breeding values for purebred and crossbred performance were converted to SNP-allele effects by breed-of-origin. Differences between purebred and crossbred, and between breeds-of-origin were evaluated by comparing percentage of variance explained by genomic regions for back fat thickness (BF), average daily gain (ADG), and residual feed intake (RFI). From ten regions explaining most additive genetic variance for crossbred performance, 1 to 5 regions also appeared in the top ten for purebred performance. The proportion of genetic variance explained by a genomic region and the estimated effect of a haplotype in such a region were different depending upon the breed-of-origin. To illustrate underlying mechanisms, we evaluated the estimated effects across breeds-of-origin for haplotypes associated to the melanocortin 4 receptor (MC4R) gene, and for the MC4Rsnp itself which is a missense mutation with a known effect on BF and ADG. Although estimated allele substitution effects of the MC4Rsnp mutation were very similar across breeds, explained genetic variance of haplotypes associated to the MC4R gene using a SNP panel that does not include the mutation, was considerably lower in one of the breeds where the allele frequency of the mutation was the lowest.

**Conclusions:**

Similar regions explaining similar additive genetic variance were observed across purebred and crossbred performance. Moreover, there was some overlap across breeds-of-origin between regions that explained relatively large proportions of genetic variance for crossbred performance; albeit that the actual proportion of variance deviated across breeds-of-origin. Results based on a missense mutation in MC4R confirmed that even if a causal locus has similar effects across breeds-of-origin, estimated effects and explained variance in its region using a commonly used SNP panel can strongly depend on the allele frequency of the underlying causal mutation.

**Electronic supplementary material:**

The online version of this article (10.1186/s12864-018-5126-7) contains supplementary material, which is available to authorized users.

## Background

In pig production, as selection is performed in purebred lines, while the final product is a crossbred animal, there is an anticipated benefit of using crossbred information for estimating breeding values of purebred for crossbred performance [1, 2]. The genetic correlation between purebred and crossbred performance (r_pc_) determines the effect of selection in the purebred animals on the rate of genetic change in the crossbred animals [3, 4]. As r_pc_ decreases, the benefit of using crossbred information increases [5, 6].

Moreover, crossbred genomic information is composed of a mosaic of genomic regions inherited from the different parental breeds (i.e., breed-of-origin). As a result, depending from which breed-of-origin an allele was inherited from, it might have different effects. These different allele effects can be due to: (1) quantitative trait loci (QTL) may be in linkage disequilibrium with different single nucleotide polymorphism (SNP) depending from which parental breed the QTL was inherited [7], (2) the functional variation that underlies the inherited QTL may have different minor allele frequencies (MAF) in different parental breeds, with the extreme case where it is not segregating in one or more breeds [8], (3) epistatic interactions in one parental breed may be different due to other genes that modify the effect of the inherited QTL in that breed [9], and above all these reasons (4) multiple and different quantitative trait nucleotides (QTN) could be underlying a QTL in different parental breeds. Therefore, the allele effect of a given SNP for purebred performance might differ from its effect for crossbred performance, and an allele of that given SNP could have a different effect on crossbred performance depending on the breed it was inherited from. Thus, SNP by genetic background interactions may be relevant when training with crossbred information to estimate breeding values of purebred animals for crossbred performance.

Several studies support that effects of SNPs may be breed-specific. Firstly, in many cases, estimated effects of SNPs in an association study for a given breed are not replicated by studies in other breeds [10–12]. Secondly, associations found in a breed often are not replicated in crossbred populations derived from this breed [13, 14]. Finally, the proportion of genetic variance in crossbred performance that is explained by each parental purebred population appears to deviate from the breed proportions [15].

With crossbreeding, SNPs can be observed in the different genetic backgrounds. Estimation of background specific effects, however, requires that the SNP alleles present in the crossbred animal are assigned to one of the parental breeds. Recently, we have developed a procedure that enables breed-of-origin assignment of alleles in three-way crossbred animals [16, 17]. Knowing the breed-of-origin, enables to estimate SNP effects for crossbred performance depending on the breed-of-origin, and to compare those within breed to estimated effects for purebred performance.

For traits with low heritability (< 0.20) and low r_pc_ (< 0.70), tracing the breed-of-origin of alleles and using this information in a genomic best linear unbiased prediction model that accounts for breed-specific SNP effects for crossbred performance (BOA model) tended to show better predictive abilities compared to models in which SNP effects are assumed to be the same across breeds [18]. This is another indication that the effect of alleles estimated for crossbred performance might be different depending upon the breed-of-origin. The objective of this study, therefore, was to investigate if the allele effect of a given SNP for crossbred performance in pigs estimated in a genomic prediction model using a commonly used SNP panel differs depending on its breed-of-origin, and how these related to estimated effects for purebred performance. For this, we estimated breed-specific SNP effects from the solutions of a BOA model. Based on previous results we chose three traits [18, 19], back fat thickness (BF) with an r_pc_ of 0.82, a heritability for crossbred performance of 0.43 and no better predictions observed when using the BOA model; average daily gain (ADG) with an r_pc_ of 0.61, a heritability for crossbred performance of 0.26 and better predictions observed when using the BOA model; and residual feed intake (RFI) with an r_pc_ of 0.62, a heritability for crossbred performance of 0.19, but not tested previously with the BOA model. To illustrate how the effect of SNP-alleles in crossbred pigs depend on their breed-of-origin, we evaluated the estimated effects across breeds-of-origin for the melanocortin 4 receptor (MC4R) gene which has a missense mutation that is known to affect BF and ADG.

## Methods

### Data

The data consisted of three purebred-based pig populations; S, LR, and LW, and one crossbred population (S (LR x LW) or S (LW x LR)). S is a synthetic sire line created as a combination of Large White and Pietrain. LR is a Landrace based dam line and LW is a Large White based dam line. All pigs were genotyped using one of the three following SNP panels: Illumina PorcineSNP60.v2 BeadChip (60 K.v2), Illumina PorcineSNP60 BeadChip (60 K), or Illumina PorcineSNP10 BeadChip (10 K). Pigs genotyped with the 60 K or 10 K chips were imputed to the 60 K.v2 panel using FImpute Version 2.2 software [20] with default parameter settings and using pedigree information. The imputation strategy was similar to Sevillano et al. [17], where each of the three purebred population, LR, LW, and S, were imputed in two steps: (1) pigs genotyped with the 10 K chip were imputed to 60 K, and (2) all pigs with 60 K data (imputed or genotyped) were imputed to 60 K.v2. This strategy was chosen because the 10 K panel shared more SNPs (8743) with the 60 K panel than with the 60 K.v2 panel (6861). For the crossbred population, imputation was done in a single step, crossbred pigs genotyped with the 10 K chip were directly imputed to 60 K.v2, because all ancestors were genotyped or already imputed to 60 K.v2.

Performance from purebred pigs were available from 52 nucleus and combined crossbred purebred system (CCPS) farms recorded from August 2005 until August 2016. Performance from crossbred pigs were available from 7 CCPS and experimental farms recorded from January 2009 until March 2016. Phenotypes for BF and ADG were measured in most of the purebred and crossbred pigs. BF for purebred pigs was measured on average at 173 days of age using an ultrasound instrument, while BF for crossbred pigs was measured on the carcass using a probe, named “capteur gras maigre” (CGM; Sydel, France), crossbred pigs were slaughtered when they achieved 120 kg (at an average age of 169 days). BF was measured approximately at the third to fourth rib from the last rib position. ADG for purebred pigs was calculated as the difference of on-test body weight at an average age of 60 days and off-test body weight at an average age of 173 days divided by the phase length. ADG for crossbred pigs was calculated as the difference of on-test body weight at an average age of 70 days of age and body weight at end of the finishing period, which was on average 120 kg, divided by the phase length. RFI was obtained as the estimated residual term from the following regression model [21]:

$$ ADFI=\upmu +{b}_1{BW}_{on}+{b}_2{BW}_{off}+{b}_3 BF+{b}_4 ADG+e, $$

in which ADFI is the average daily feed intake, μ is the mean, *BW*_*on*_ is the on-test body weight, *BW*_*off*_ is the off-test body weight, BF and ADG are the previously described traits, *b*_*1*_, *b*_*2*_, *b*_*3*_, and *b*_*4*_ are the linear coefficients of the regression on covariates, and *e* is the RFI. The numbers of available genotypes and phenotypes per trait and per population are summarized in Table 1. For all phenotyped pigs, four generations of pedigree information were included for analysis.

**Table 1:** Number of genotypes and phenotypes available per trait and per population

Population	Genotypes	BF/ADG	RFI
S	8079	7547	2102
LR	5233	3288	56
LW	15,727	12,794	1133
Crossbred	3352	2816	2695
Total	32,391	26,445	5986

*BF* = back fat thickness, *ADG* = average daily feed intake, and, *RFI* = residual feed intake

### Estimation of SNP-allele effects

SNP-allele substitution effects were estimated using best linear unbiased predictions (BLUP) similar to Wang et al. [22]. However, instead of using a single-step BLUP, we used a genomic BLUP (GBLUP) with breed-specific partial relationship matrices, i.e., BOA model [18]. With this approach, genomic estimated breeding values (GEBV) for purebred and crossbred performance could be calculated, and posteriorly converted to SNP-allele effects by breed-of-origin. SNP-allele effects were derived using the following steps:

1. Determine breed-of-origin of alleles to calculate breed-specific partial relationship matrices, **G**^(**S**)^, **G**^(**LR**)^, and **G**^(**LW**)^.
2. Calculate GEBVs for purebred and crossbred performance using the BOA model.
3. Back-solve SNP-allele effects for purebred and crossbred performance from GEBVs.
4. Calculate proportion of variance explained by non-overlapping blocks of SNPs.

#### Inference of the breed-of-origin of alleles

To infer breed-of-origin of alleles in crossbred pigs we used the breed of origin of alleles approach (BOA approach) developed by Vandenplas et al. [16] and assuming the parameter settings recommended by Sevillano et al. [17]. The BOA approach consisted of three steps: (1) Phasing the haplotypes of both purebred and crossbred pigs with AlphaPhase1.1 software [23]. Phasing was performed using pedigree, and using nine combinations of haplotypes length and each combination was run both considering “Offset” and “NotOffset” modes, the “Offset” mode shifts the start of the cores to halfway along the first core, creating 50% overlaps between cores. These settings allowed each allele to be considered 18 times through different haplotypes of variable length. (2) Determining the unique haplotypes among the purebred pigs. For assigning a breed-of-origin to a haplotype, at least 80% of its copies were required to be observed in a specific breed. (3) Assigning the breed-of-origin for each allele carried on the haplotypes of crossbred pigs based on the knowledge of the breed-of-origin of the haplotypes, on the zygosity (i.e., homozygosity or heterozygosity) of the locus, and on the breed composition of the crossbred. Alleles that were not assigned a breed-of-origin were set to missing. SNPs for which the paternal or maternal allele were assigned a breed-of-origin in less than 90% of the cases were removed. Crossbred pigs with assigned breed-of-origin for less than 90% of their genome were removed. If an allele was observed less than 5 times in any of the breeds-of-origin, the corresponding SNP was also removed from the final set of SNPs. The final SNP set for subsequent analyses consisted of 41,557 SNPs. All populations were analysed with the same set of SNPs.

#### Model with three breed-specific partial relationship matrices

To account for breed-specific effect of alleles, a 4-trait animal model (i.e., S trait, LR trait, LW trait and crossbred trait) with three breed-specific partial relationship matrices (**G**^(**S**)^**,G**^(**LR**)^**,G**^(**LW**)^) was fitted (i.e., BOA model) [18]. The three breed-specific partial relationship matrices, **G**^(**S**)^, **G**^(**LR**)^, and **G**^(**LW**)^, were built using the breed-of-origin of phased alleles in crossbred pigs and the first method from VanRaden [24]. The breed-specific partial relationship submatrices are defined, considering e.g. the breed S origin, as:

$$ {\displaystyle \begin{array}{l}{\mathbf{G}}_{\mathbf{S},\mathbf{S}}=\left({\mathbf{M}}^{\mathbf{S}}-21{\mathbf{p}}^{\mathbf{S}\hbox{'}}\right){\left({\mathbf{M}}^{\mathbf{S}}-21{\mathbf{p}}^{\mathbf{S}\hbox{'}}\right)}^{\hbox{'}}{\mathrm{F}}^{-1},\\ {}{\mathbf{G}}_{\mathbf{S},\mathbf{CB}}=\left({\mathbf{M}}^{\mathbf{S}}-21{\mathbf{p}}^{\mathbf{S}\hbox{'}}\right){\left({\mathbf{M}}^{\mathbf{CB}}-1{\mathbf{p}}^{\mathbf{S}\hbox{'}}\right)}^{\hbox{'}}{\mathrm{F}}^{-1},\mathrm{and}\\ {}{\mathbf{G}}_{\mathbf{CB},\mathbf{CB}}=\left({\mathbf{M}}^{\mathbf{CB}}-1{\mathbf{p}}^{\mathbf{S}\hbox{'}}\right){\left({\mathbf{M}}^{\mathbf{CB}}-1{\mathbf{p}}^{\mathbf{S}\hbox{'}}\right)}^{\hbox{'}}{\mathrm{F}}^{-1},\end{array}} $$

where **M**^**S**^ is a matrix containing breed-specific allele content for purebred S pigs (coded as 0, 1, or 2). **M**^**CB**^ is a matrix containing breed-specific allele content for crossbred pigs (coded as 0, or 1), so that alleles not assigned a breed-of-origin were set to missing, meaning that they had an entry of zero in the centred matrix represented by (**M**^**CB**^ − **1p**^**S**′^); **p**^**S**^ is the vector of breed S specific frequencies of the counted allele (${p}_j^s\Big)$, where ${p}_j^s$ was calculated across S and crossbred pigs by counting the occurrences of alleles originating from the S breed and coded as 1, divided by the total number of S alleles in the S breed and crossbred on locus *j*. Finally, the scaling factor was defined as $\mathrm{F}={\sum}_j2{p}_j^S\left(1-{p}_j^S\right)$. The breed-specific partial relationship submatrices **G**^(**LR**)^ and **G**^(**LW**)^ are defined similarly to **G**^(**S**)^. Other effects in the model included fixed effects partially depending on the trait (Table 2), and random common litter effects. The BOA model was implemented in the MiXBLUP software [25]. To estimate variance components we used the same BOA model in the ASReml software [26], after reducing each of the purebred populations to 3500 pigs most closely related to the crossbred population.

**Table 2:** Fixed effects used in the models for each trait for purebred and crossbred pigs

Trait	Population	Fixed effects
BF	Purebred	farm ∗ breed ∗ sex + b_a_ × off − test BW
	Crossbred	trial + farm ∗ sex + b_b_ × hot carcass weight
ADG	Purebred	farm ∗ breed ∗ sex + b_c_ × birth weight
	Crossbred	trial + farm ∗ sex + b_c_ × birth weight
RFI	Purebred	farm ∗ breed ∗ sex + b_d_ × on − test BW
	Crossbred	trial + farm ∗ breed ∗ sex + b_d_ × on − test BW

*BF* = back fat thickness, *ADG* = average daily gain, and *RFI* = residual feed intake

b_a_, b_b_, b_c_, b_d_, are regression coefficients for off-test BW, hot carcass weight, birth weight, and on-test BW, respectively

#### Back-solve SNP-allele effects from GEBV

GEBV of purebred S pigs for purebred performance (${\widehat{\mathbf{a}}}_{\mathbf{S}}$) were converted to SNP-allele effects (${\hat{\upalpha}}_{\mathrm{s}}$), considering that:

$$ {\widehat{\mathbf{a}}}_{\mathbf{S}}={\mathbf{W}}^{\mathbf{S}}{\widehat{\boldsymbol{\upalpha}}}_{\mathbf{s}} $$

where **W**^**S**^ contains centered genotypes, which can be obtained respectively by:

$$ {\displaystyle \begin{array}{c}{\mathbf{W}}^{\mathbf{S}}=\left({\mathbf{M}}^{\mathbf{S}}-21{\mathbf{p}}^{\mathbf{S}\hbox{'}}\right),\mathrm{and}\\ {}\kern0ex \end{array}} $$

$$ {\widehat{\boldsymbol{\upalpha}}}_{\mathbf{s}}={\mathbf{W}}^{\mathbf{S}\hbox{'}}{\left({\mathbf{W}}^{\mathbf{S}}{\mathbf{W}}^{\mathbf{S}\hbox{'}}\right)}^{-1}{\widehat{\mathbf{a}}}_{\mathbf{S}}={\mathrm{F}}^{-1}{\mathbf{W}}^{\mathbf{S}\hbox{'}}{\mathbf{G}}^{\left(\mathbf{S}\right)-1}{\widehat{\mathbf{a}}}_{\mathbf{S}} $$

The SNP-allele effects for crossbred performance and for the other purebred populations were calculated similarly.

#### Variance proportion explained by SNP regions

Under a back-solving approach, all SNPs are considered simultaneously in the model, therefore, the effect of a QTL is likely distributed across all SNPs that have a nonrandom association with the QTL. For this reason, it is recommended to calculate the proportion of variance explained by a group of SNPs in nonrandom association instead of reporting effects of single SNPs [7]. Groups of SNPs in nonrandom association will hereafter be called LD blocks. LD blocks were built per breed-of-origin, therefore, nonrandom association between alleles at two loci was tested in the crossbred population between all pair of loci coming from the same breed-of-origin. Significant nonrandom association between alleles at two loci was tested with Fisher’s exact test on a contingency table made for counts of the four gametic types at the two loci [27]. If statistical significant nonrandom association is detected (*P*-value< 0.01), then it can be concluded that the coefficient of linkage disequilibrium, D, is significantly different from zero and that pair of loci are in linkage disequilibrium [28]. Breakpoints between LD blocks were defined when D between two consecutive SNPs was not significantly different from zero. Estimation of D and Fisher’s exact test was performed using the Arlequin software [29].

Percentage of genetic variance explained by the *i*-th LD block was calculated as in Wang et al. [22]:

$$ \frac{\mathrm{Var}\left({\mathrm{a}}_i\right)}{\upsigma_{\mathrm{a}}^2}\times \frac{{\mathrm{x}}_{\mathrm{n}}}{{\mathrm{n}}_i}\times 100\%=\frac{\mathrm{Var}\left(\sum \limits_{\mathrm{j}=1}^{\mathrm{n}}{\mathrm{z}}_{\mathrm{j}}{\widehat{\upalpha}}_{\mathrm{j}}\right)}{\upsigma_{\mathrm{a}}^2}\times \frac{{\mathrm{x}}_{\mathrm{n}}}{{\mathrm{n}}_i}\times 100\%, $$

where a_i_ is genetic value of the *i*-th LD block, ${\upsigma}_{\mathrm{a}}^2$ is the total genetic variance, z_j_ is a vector of genotypes of the *j*-th SNP for all purebred individuals of the same breed, ${\widehat{\upalpha}}_{\mathrm{j}}$ is the estimated effect of the *j*-th SNP within the *i*-th LD block that contains *n* SNPs, x_n_is the mean number of SNPs across LD blocks and n_*i*_is the number of SNPs of the *i*-th LD block. With the back-solving approach we can identify peaks that explain the most variance, in our case, we took the top 10 LD blocks for comparison across scenarios.

### Candidate genes

Putative candidate genes within the top 10 LD blocks and in the neighbouring upstream and downstream 1-Mb regions were identified based on the Sscrofa11.1 genome assembly, using the NCBI Map Viewer (https://www.ncbi.nlm.nih.gov/genome/gdv/?org=sus-scrofa) and based on literature.

### MC4R

To illustrate the mechanisms underlying breed-of-origin specific estimated SNP effects, we investigated the estimated effects across breeds-of-origin for haplotypes associated to the MC4R gene, and the allele substitution effects for the MC4Rsnp itself. The MC4R gene has a missense mutation that is known to have a strong effect on BF and ADG [30]. The genotypes at the MC4Rsnp were available for 4996 S, 1363 LR, 7663 LW, and 1478 crossbred pigs. The MC4Rsnp is biallelic (A|G) and located in the MC4R gene at 160,772,887 bp of the SSC1; allele A is the mutant allele (hereafter denoted as allele *m*) and allele G is the wild type allele (hereafter denoted as allele *w*). The MC4Rsnp was imputed in pigs that were not genotyped for it and the breed-of-origin of both alleles were inferred with the BOA approach. After quality control we had information for 7469 S, 3257 LR, 12707 LW, and 2763 crossbred pigs. Allele frequencies of the MC4Rsnp were computed in each of the purebred populations and in the crossbred population considering breed-of-origin. In order to build LD blocks that co-segregate with the MC4R gene, linkage disequilibrium was tested between the alleles of MC4Rsnp and the alleles from all the other loci in the SSC1 of the crossbred population [27]. Unlike the LD blocks previously built, breakpoints to define the MC4R LD blocks were not defined when D between two consecutive SNPs was not significantly different from zero, but when D between the MC4Rsnp and any of the other SNPS in the SSC1 was not significantly different from zero. The effect of each haplotype present in the LD block that co-segregate with the MC4R gene was calculated per breed-of-origin for crossbred performance for ADG.

To enable comparison to the estimated haplotype effects we also estimated the effect of the MC4Rsnp itself. The effect was estimated with the software ASReml [13] by applying the following model:

$$ {ADG}_{ij}=\mu +{b}_S MC4{Rsnp}_S+{b}_{LR} MC4{Rsnp}_{LR}+{b}_{LW} MC4{Rsnp}_{LW}+{c}_i^2+{u}_j+{e}_{ij}, $$

where *ADG*_*ij*_ was the pre-corrected ADG phenotype of crossbred pig *j*, ADG phenotypes were corrected for fixed effects listed in Table 2; *MC*4*Rsnp*_*S*_, *MC*4*Rsnp*_*LR*_, and *MC*4*Rsnp*_*LW*_ were the centered allele content of MC4Rsnp (0 or 1) of crossbred *j* for breed-of-origin S, LR, and LW, respectively; *b*_*S*_, *b*_*LR*_, and *b*_*LW*_ were the unknown allele substitution effect of MC4Rsnp for breed-of-origin S, LR, and LW, respectively; ${c}_i^2$ was the random effect of common litter *i*, assumed to be normally distributed ~ *N*(0, **I**${\sigma}_c^2$), where **I** was an identity matrix and ${\sigma}_c^2$ was the unknown variance between litters; *a*_*j*_ was the random additive genetic effect of crossbred *j* assumed to be normally distributed ~ *N*(0, **A**${\sigma}_u^2$), where **A** was a known matrix of additive genetic relationship among pigs (pedigree-based) and ${\sigma}_u^2$ was the genetic variance between pigs that was estimated in the BOA model; and *e*_*ij*_ was the random residual effect assumed to be normally distributed ~ *N*(0, **I**${\sigma}_e^2$), where ${\sigma}_e^2$ was the unknown residual variance.

## Results

### Heritabilities and genetic correlations

Estimated variance components and standard errors for BF, ADG, and RFI using the BOA model are shown in Table 3. Estimates of crossbred heritability tended to be larger than estimates of purebred heritability. The lowest heritability for crossbred performance was observed for ADG (0.29), while BF and RFI showed similar heritabilities of 0.41 and 0.40, respectively. The lowest r_pc_ was observed for RFI (0.37–0.60), followed by ADG (0.60–0.69), while BF showed the highest r_pc_ (0.71–0.89). Because of the limited number of RFI records from LR pigs, genetic parameters estimated in this population had very high standard errors, therefore, estimates are not shown and were not further used in this study.

**Table 3:** Heritability estimates for purebred (${h}_{PB}^2$) and crossbred (${h}_{CB}^2$) performance, and genetic correlation between performance in purebred and crossbred (*r*_*PC*_)

Trait	Breed	${h}_{PB}^2$	${h}_{CB}^2$	*r*_*PC*_
BF	S	0.31 (0.02)	0.41 (0.04)	0.80 (0.07)
	LR	0.33 (0.03)		0.71 (0.10)
	LW	0.34 (0.03)		0.89 (0.09)
ADG	S	0.09 (0.02)	0.29 (0.04)	0.69 (0.12)
	LR	0.22 (0.02)		0.60 (0.16)
	LW	0.20 (0.02)		0.68 (0.13)
RFI	S	0.15 (0.03)	0.40 (0.05)	0.37 (0.14)
	LR	–		–
	LW	0.61 (0.05)		0.60 (0.18)

*BF* = back fat thickness, *ADG* = average daily gain, and, *RFI* = residual feed intake

### Proportion of genetic variance explained by a region

The number and size of the LD blocks are shown per breed-of-origin in Table 4. The LD blocks coming from the S breed-of-origin were on average the longest (7.1 SNPs), followed by the LD blocks coming from the LW breed-of-origin (6.4 SNPs), while the LD blocks coming from the LR breed-of-origin were on average the shortest (5.3 SNPs).

**Table 4:** Description of LD blocks built per breed-of-origin

Breed-of-origin	Number of blocks	Length (number of SNPs)		
		Mean	Min	Max
S	5516	7.1	1	115
LR	7495	5.3	1	56
LW	6296	6.4	1	86

**Fig. 1:**
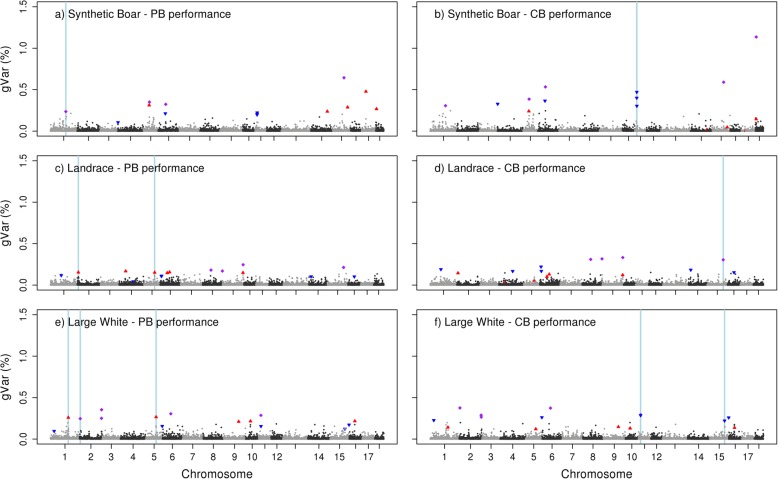
Proportion of genetic variance for back fat thickness explained by each LD block. Observed in S (**a**), LR (**c**), and LW (**e**) for purebred performance (PB) and when alleles originate from S (**b**), LR (**d**), or LW (**f**) for crossbred performance (CB). Top 10 LD blocks explaining most variance for PB (red ▲), and top 10 LD blocks explaining most variance for CB performance (blue ▼). LD blocks belonging to the top 10 in both, PB and CB performance (purple ♦). Regions explaining the variance for PB in more than one breed or explaining the variance for CB in more than one breed-of-origin (light blue strip)

**Fig. 2:**
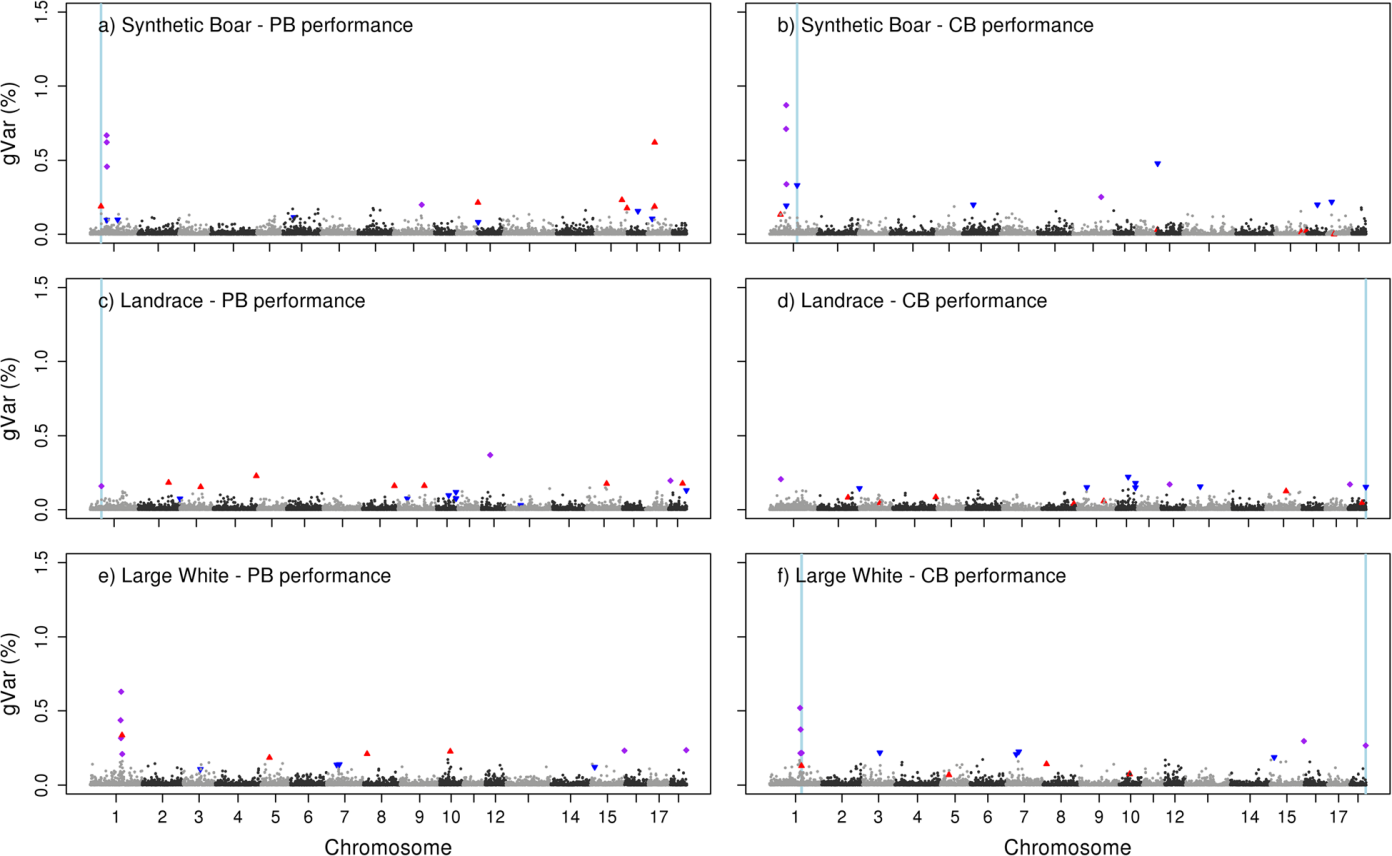
Proportion of genetic variance for average daily gain explained by each LD block. Observed in S (**a**), LR (**c**), and LW(**e**) for purebred performance (PB) and when alleles originate from S (**b**), LR (**d**), or LW (**f**) for crossbred performance (CB). Top 10 LD blocks explaining most variance for PB (red ▲), and top 10 LD blocks explaining most variance for CB performance (blue ▼). LD blocks belonging to the top 10 in both, PB and CB performance (purple ♦). Regions explaining the variance for PB in more than one breed or explaining the variance for crossbred in more than one breed-of-origin (light blue strip)

**Fig. 3:**
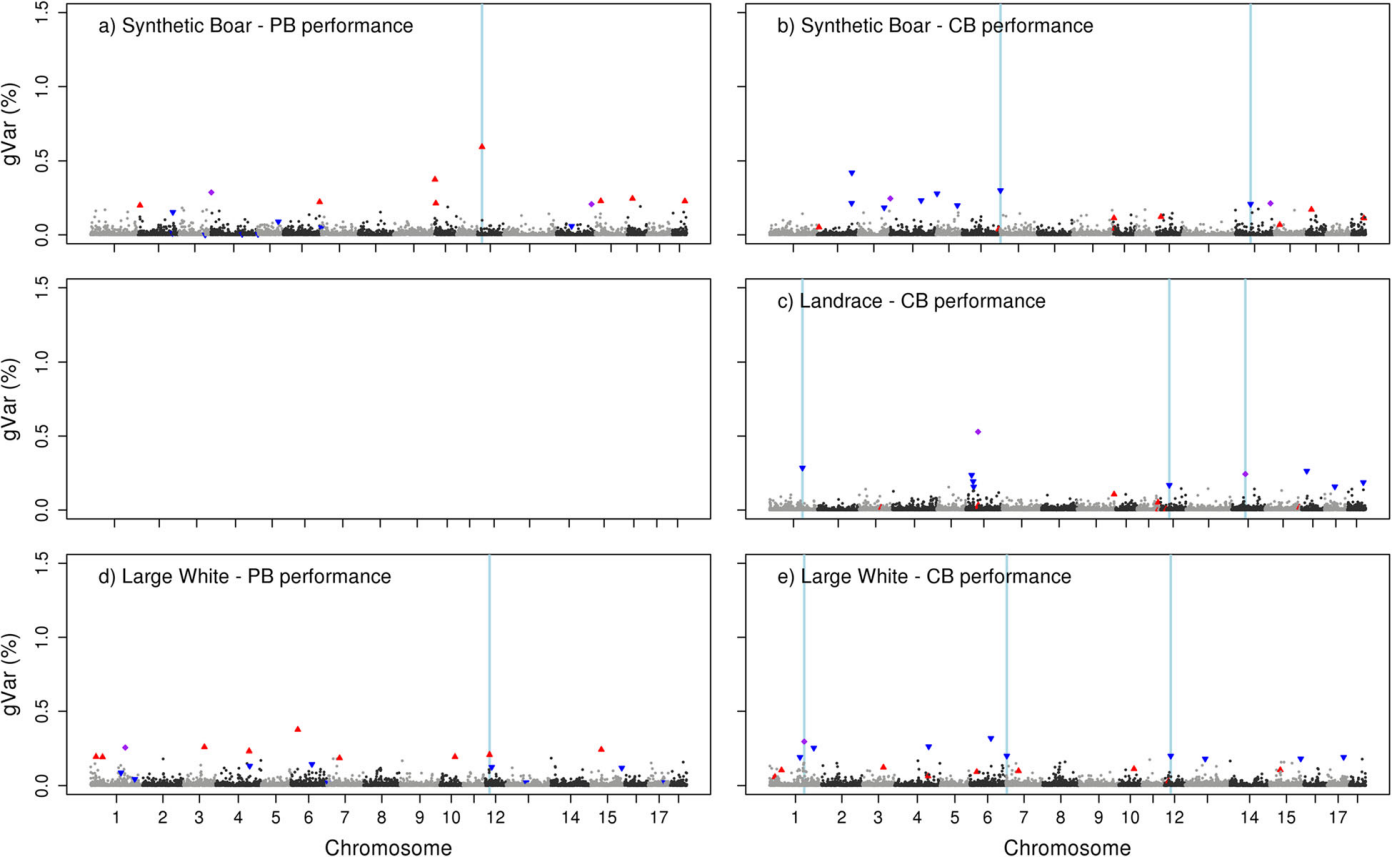
Proportion of genetic variance for residual feed intake explained by each LD block. Observed in S (**a**), and LW (**d**) for purebred performance (PB) and when alleles originate from S (**b**), LR (**c**), or LW(**e**) for crossbred performance (CB). Top 10 LD blocks explaining most variance for PB (red ▲), and top 10 LD blocks explaining most variance for CB performance (blue ▼). LD blocks belonging to the top 10 in both, PB and CB performance (purple ♦). Regions explaining the variance for PB in more than one breed or explaining the variance for CB in more than one breed-of-origin (light blue strip). *Because of the limited number of RFI records from LR pigs, genetic parameters estimated for LR breed – PB performance had high SE, therefore, estimates are not shown

Figures 1, 2 and 3 show for each breed genetic variances for all LD blocks for purebred and crossbred performance for BF, ADG, and RFI, respectively. Depending on the breed and across traits, the proportion of genetic variance jointly explained by the top 10 LD blocks with most explained genetic variance ranged, across breeds and traits, from 1.73 to 4.51% for purebred performance and from 1.71 to 4.51% for crossbred performance (Table 5). Depending on the trait, and considering that the haploid genome of the domesticated pig is estimated to be 2800 Mb, the top 10 LD blocks covered at least 0.19% and at the most 0.47% of the genome. Proportion of genetic variance and position of each of the top 10 LD blocks for purebred and crossbred performance by breed are detailed in Additional files 1, 2 and 3 for BF, ADG and RFI, respectively.

**Table 5:** Percentage of genetic variance explained by top ten LD blocks for purebred and crossbred (CB) performance

%^a^	BF	ADG	RFI
Purebred			
gVar S	4.51	3.57	2.80
gVar LR	1.73	1.81	–
gVar LW	2.61	3.00	2.33
CB,breed-of-origin			
gVar CB,S	4.51	3.80	2.50
gVar CB,LR	2.35	1.71	2.42
gVar CB,LW	2.85	2.71	2.28

^a^Percentage of genetic variance for purebred performance by breed and for crossbred (CB) performance by breed-of-origin

*BF* = back fat thickness, *ADG* = average daily gain, and, *RFI* = residual feed intake

LD blocks that appeared for both purebred and crossbred performance in the top 10 with most explained genetic variance, are shown per breed-of-origin in Table 6. Depending on the breed, the number of LD blocks from the top 10 that appeared for both purebred and crossbred performance, was 4 to 5 for BF, 3 to 6 for ADG, and at most one for RFI. For the LD blocks that appeared for both purebred and crossbred performance in the top 10, the percentage of genetic variance they explained for both purebred and crossbred performance was quite similar.

**Table 6:** LD blocks in common^a^ between crossbred and purebred performance per breed-of-origin

Trait	S_PB_-S_CB_			LR_PB_-LR_CB_			LW_PB_-LW_CB_		
	Position Chr:Mb	gVar PB	gVar CB	Position Chr:Mb	gVar PB	gVar CB	Position Chr:Mb	gVar PB	gVar CB
BF	SSC1:158,9-160,2	0.24	0.31	SSC8: 60,6 – 64,0	0.18	0.31	SSC2: 9,8 – 10,5	0.25	0.38
	SSC5: 30,1 – 30,8	0.35	0.39	SSC9: 0–0,6	0.17	0.32	SSC2: 144,6 – 144,7	0.25	0.29
	SSC6: 47,8 – 49,9	0.32	0.53	SSC9: 128,3 – 128,9	0.25	0.33	SSC2: 144,8 – 145,1	0.35	0.26
	SSC15: 102,3 – 104,5	0.64	0.59	SSC15: 119,3 – 119,8	0.21	0.30	SSC6: 77,5 – 78,4	0.31	0.37
	SSC18: 10,1 – 10,6	1.62	1.14				SSC11: 7,6 – 7,9	0.29	0.29
ADG	SSC1: 50,8 – 51,4	0.67	0.71	SSC1: 24,2–25,6	0.16	0.21	SSC1: 148,2 – 150,4	0.44	0.52
	SSC1: 51,5 – 52,7	0.62	0.87	SSC12: 16,5 – 17,3	0.37	0.17	SSC1:152,4 – 153,1	0.32	0.21
	SSC1: 53,8 – 54,1	0.46	0.34	SSC18: 9,8 – 9,9	0.20	0.17	SSC1: 154,1 – 155,5	0.63	0.37
	SSC9: 76,1–78,8	0.20	0.25				SSC1: 160,9 – 162,4	0.21	0.22
							SSC15: 132,5 – 132,9	0.23	0.30
							SSC18: 53,2 – 53,9	0.23	0.27
RFI	SSC14: 125,3 – 125,9	0.21	0.21				SSC1: 183,7 – 184,8	0.25	0.29

^a^Considering only the top 10 LD blocks explaining most of the genetic variance for purebred or crossbred performance

*gVar PB* = percentage of genetic variance explained by a LD block for purebred performance

*gVar CB* = percentage of genetic variance explained by a LD block for crossbred performance

*BF* = back fat thickness, *ADG* = average daily gain, and *RFI* = residual feed intake

*S*_*PB*_ = PB performance for S breed, LR_PB_ = PB performance for LR breed, and LW_PB_ = PB performance for LW breed

*S*_*CB*_ = CB performance for S breed-of-origin, *LR*_*CB*_ = CB performance for LR breed-of-origin, and *LW*_*CB*_ = CB performance for LW breed-of-origin

As LD blocks across breed-of-origin were not the same, because of different patterns of linkage disequilibrium, comparisons across breeds for crossbred performance were done regarding whether the top 10 LD blocks across breeds overlapped or were less than 1-Mb distance apart (Table 7). These regions can be observed in Figs. 1, 2 and 3 in light blue. From the top 10 LD blocks, at most, one region in common was observed between breeds for crossbred performance per trait. For both BF and ADG performance in crossbred, there were no common regions between the top 10 LD blocks from S breed-of-origin and the top 10 LD blocks from LR breed-of-origin.

**Table 7:** LD blocks in common^a^ across breed-of-origin for crossbred performance

Trait	S_CB_-LR_CB_		S_CB_-LW_CB_		LR_CB_-LW_CB_	
	Chr:Mb_S_ Chr:Mb_LR_	gVar S gVar LR	Chr:Mb_S_ Chr:Mb_LW_	gVar S gVar LW	Chr:Mb_LR_ Chr:Mb_LW_	gVar LR gVar LW
BF			SSC11: 7,6–9.9	1.18	SSC15: 119,3 – 119,8	0.30
			SSC11: 7,6 – 9,4	0.58	SSC15: 118,2 – 118,8	0.22
ADG			SSC1: 158,9 – 160,2	0.33	SSC18: 54,3 – 54,7	0.15
			SSC1: 160,9 – 162,4	0.22	SSC18: 53,2 – 53,9	0.27
RFI	SSC14: 46,2 – 48,0	0.21	SSC7: 2,2 – 2,8	0.30	SSC1: 183,9 – 185,0	0.53
	SSC14: 45,8 – 47,8	0.19	SSC7: 2,6 – 3,0	0.20	SSC1: 183,7 – 184,8	0.29

^a^Because LD blocks are different between breed-of-origin, comparisons were done regarding whether the top 10 LD blocks explaining most of the genetic variance across breed-of-origin overlapped or were less than 1-Mb distance apart

*BF* = back fat thickness, *ADG* = average daily gain, and *RFI* = residual feed intake

*S*_*CB*_ = CB performance for S breed-of-origin, *LR*_*CB*_ = CB performance for LR breed-of-origin, and *LW*_*CB*_ = CB performance for LW breed-of-origin

*gVar* = percentage of genetic variance explained by a LD block according to the breed-of-origin

A similar comparison was made across breeds for purebred performance. For BF, there was one common region between the top 10 LD blocks from S and LW and there were two common regions between the top 10 LD blocks from LR and LW. For ADG, there was one common region between the top 10 LD blocks from S and LR. For RFI, comparisons could be only made between S and LW because the SNP-allele effects for the LR population were not estimated, there was one common region between the top 10 LD blocks from S and LW. These regions can be observed in Figs. 1, 2 and 3 in light blue.

### Candidate genes

Putative candidate genes within the top 10 LD blocks either for purebred or crossbred performance and in the neighbouring upstream and downstream 1-Mb regions were identified based on the Sscrofa11.1 genome assembly and based on literature. The MC4R was identified as a candidate gene for ADG and BF. The MC4R gene was previously associated with feed intake and growth rate in pigs, as well as with BF [30–33]. The MC4R gene controls energy balance [34]. MC4R are broadly distributed in the central neuronal system and an agonist stimulation at MC4R leads to a decrease in feed intake and loss of body weight [34]. The MC4R gene is located on SSC1 at 160,771,802 – 160,774,335 bp. For S, the gene was contained in a LD block located at 160.2–161.4 Mb. However, the LD block in the previous position (158.9–160.2 Mb) was in the top 10 with most explained genetic variance for crossbred performance for ADG and BF. This LD block explained a large variance for purebred performance for BF although it did not make it into the top 10 LD blocks. For LR, this region seems not to contain any QTL. For LW, the MC4R gene was located in an LD block located at 160.2–160.7 Mb. However, a second LD block, located immediately before (159.2–160.2 Mb) was in the top 10 with most explained genetic variance for purebred performance for ADG and BF. Additionally, a third LD block, located immediately after (160.9–162.4 Mb) was in the top 10 with most explained genetic variance for both in purebred and crossbred performance for ADG.

The StAR-related lipid transfer domain containing 13 (STARD13) was identified as a candidate gene for BF. The STAR gene family is involved with lipids and lipid hormones binding to be exchanged between biological membranes [35]. STARD13 seems to regulate FOS gene expression, which is a gene functionally related with intramuscular fatty acid composition [36]. The STARD13 gene is located on SSC11 at 9,496,111 – 9,760,394 bp. For S, the gene was located in a LD block located at 8.9–9.9 Mb. This LD block was in the top 10 with most explained variance for crossbred performance for BF. Two contiguous LD blocks (7.6–7.9 Mb and 8.0–8.8 Mb) were also in the top 10 with most explained variance for crossbred performance for BF. These three LD blocks explained a relatively large part of the variance for purebred performance for BF although they did not make it to the top 10 LD blocks. For LR, this region does not seem to contain any QTL. For LW, the STARD13 gene overlapped one LD block (9.5–9.7 Mb). However, the LD blocks in the previous positions (7.6–7.9 Mb and 8.0–9.4 MB) were in the top 10 with most explained variance for BF performance in purebred and crossbred, and crossbred, respectively.

The porcine insulin-like growth factor binding protein (IGFBP-5) was also identified as a candidate gene for BF. IGFBP-5 is a focal regulatory factor during the development of several key cell types, e.g., myoblasts and neural cells [37]. The IGFBP-5 gene might be involved in intramuscular fat development in cattle [38], and was also associated with fat deposition in pigs [39]. The IGFBP-5 gene is located on SSC15 at 118,860,219 – 118,879,384 bp. For S, this region does not seem to contain any QTL. For LR, the gene was contained in a LD block located at 118.6–118.9 Mb. However, the LD block in a following position (119.3–119.8 Mb) was in the top 10 with most explained variance for purebred and crossbred performance for BF. For LW, the gene was contained in a LD block located at 118.8–119.0 Mb. However, the LD block in the previous position (118.2–118.8 Mb) was in the top 10 with most explained variance for crossbred performance for BF.

We did not identify any candidate gene for RFI. For RFI, there are few GWAS studies in pigs and they all revealed different regions associated with this trait [32, 33, 40, 41]. RFI is a complex trait and the biology behind it seems difficult to unravel, as we were unable to find LD blocks explaining a large percentage of genetic variance or patterns across purebred and crossbred performance within the same breed.

### MC4R

From all evaluated candidate genes, only for the MC4R gene the underlying causal mutation is known. Allele frequencies of this MC4Rsnp were quite similar between observed frequencies in purebred compared to crossbred pigs, but considerable differences were observed between breeds within the purebred or between breeds-of-origin within the crossbred (Table 8). In the S population and among alleles originating from S in the crossbred population, the *m* allele is highly prevalent (0.81–0.85), whereas in the LR population or among alleles originating from LR in the crossbred population, the *m* allele is almost absent (0.06–0.11).

**Table 8:** Frequency of MC4Rsnp alleles^a^ in purebreds and in crossbreds (CB) within breed-of-origin

	*m*	*w*
Purebred		
S	0.85	0.15
LR	0.06	0.94
LW	0.39	0.61
CB,breed-of-origin^b^		
CB,S	0.81	0.19
CB,LR	0.12	0.88
CB,LW	0.44	0.56

^a^*m* is associated with the mutant allele and allele *w* is associated with the wild allele of MC4R

^b^Expressed as frequency within each breed-of-origin

For S breed-of-origin, the MC4Rsnp was in LD with 31 flanking loci, which resulted in a LD block from 158.9 to 161.5 Mb. For LR breed-of-origin, the MC4Rsnp was in LD with 49 flanking loci, which resulted in a LD block from 158.8 to 163.3 Mb. For LW breed-of-origin, the MC4Rsnp was in LD with 42 flanking loci, which resulted in a LD block from 158.9 to 162.6 Mb. For comparison across breed-of-origin, we only considered the overlapping SNPs across the three LD blocks which resulted in a block of 31 SNPs (158.9–161.5 Mb). It is worthwhile noting that this MC4R based block contains the LD block spanning 158.9–160.2 Mb that was identified to be associated with ADG and BF in S and the LD block spanning 159.2–160.2 Mb associated with ADG and BF in LW. The block contained 74 different haplotypes, each unique haplotype was always exclusively co-segregating either the *m* or *w* allele of MC4Rsnp. The only exception was a haplotype that was observed in 83 crossbred pigs originating from S, in 260 crossbred pigs originating from LR, and in 1993 crossbred pigs originating from LW. This haplotype carried the *m* allele for all these crossbred pigs, except for two who received the haplotype from S and carried the *w* allele. These two cases, however, may simply be genotyping errors and were not used further for the MC4R analysis. Therefore, after including the MC4Rsnp in the LD block we still observed 74 different haplotypes. From the 74 haplotypes, 44 were observed in the S breed-of-origin, 19 in the LR breed-of-origin and 31 in the LW breed-of-origin.

**Fig. 4:**
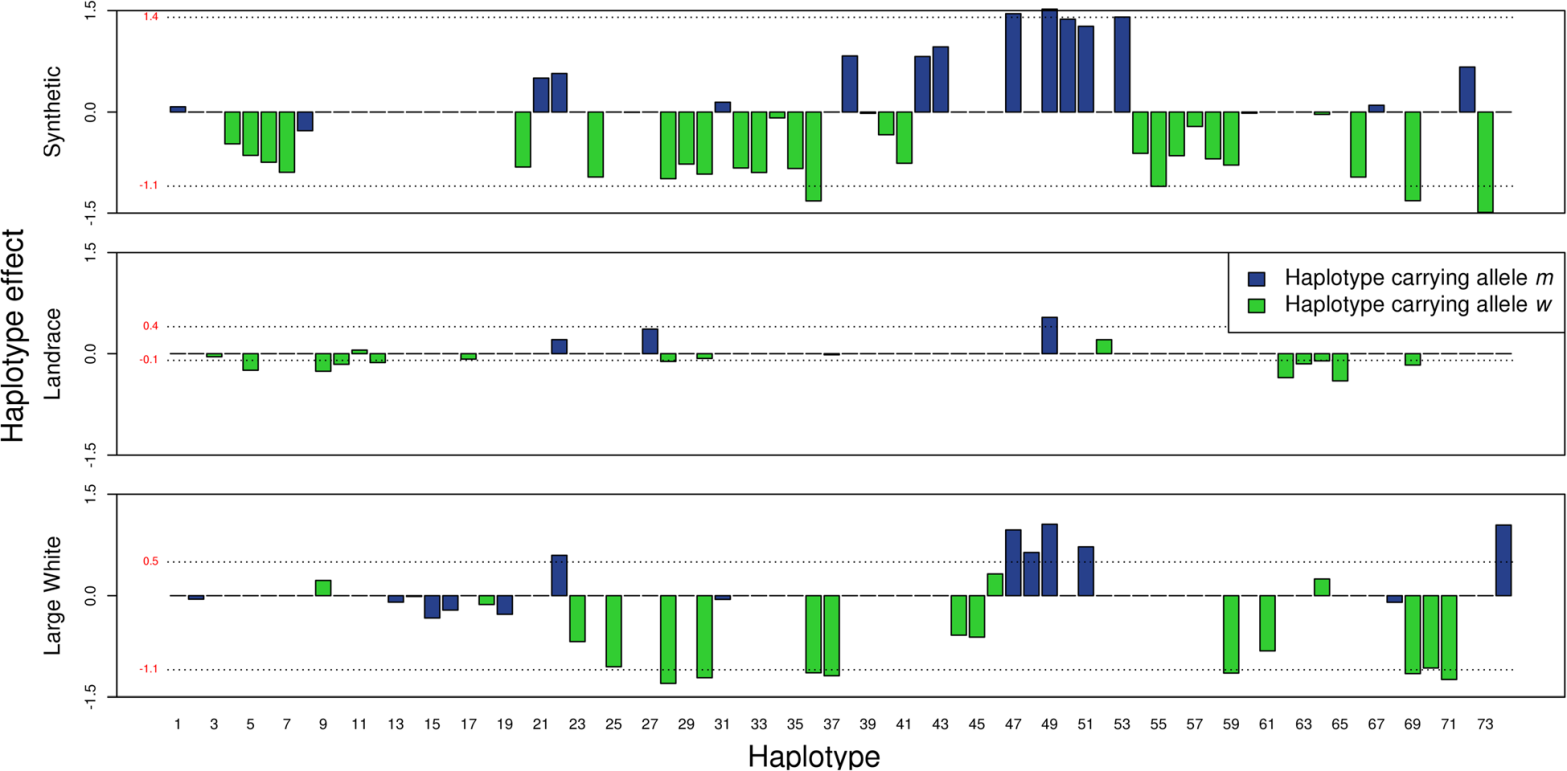
Haplotypes effect on average daily gain (g/d) per breed-of-origin. From the 74 haplotypes observed in the LD block associated with the MC4R gen. Average effects of the *m* and *w* allele, weighted according to the haplotype frequencies, are shown as red numbers

**Fig. 5:**
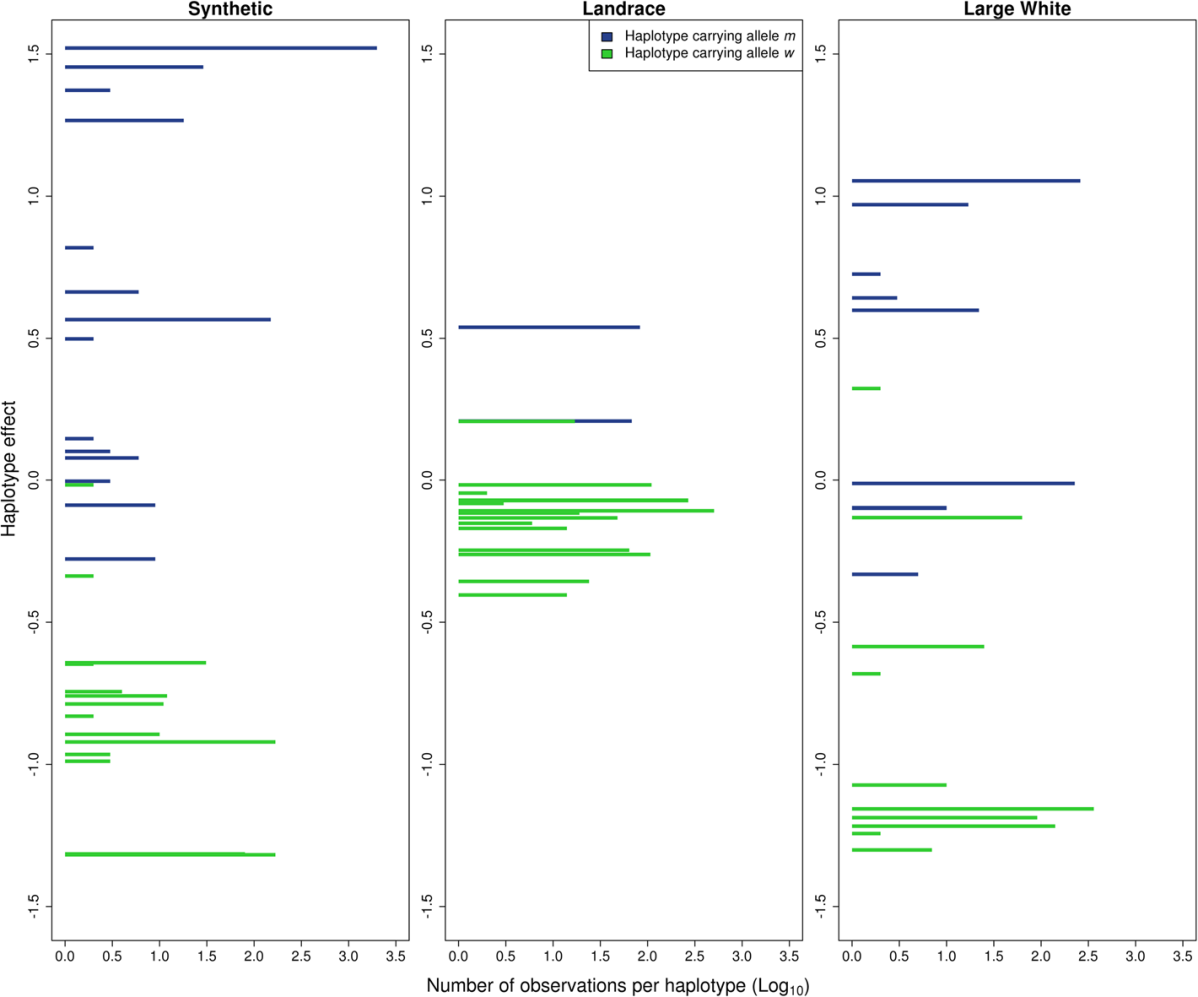
Number of observations (Log_10_) of each of the 74 haplotypes. Number of observations are presented according to the effect of the haplotype on average daily gain (g/d). From the 74 haplotypes observed in the LD block associated with the MC4R gen

In Fig. 4, the effect of each haplotype that co-segregates with the MC4R gene is shown per breed-of-origin for crossbred performance for ADG. Within breed-of-origin, haplotypes co-segregating with the *m* allele had different effects compared to haplotypes co-segregating with the *w* allele (T-test, *P*-value < 0.05). Haplotypes co-segregating with the *m* allele, in general, had a positive effect, while haplotypes co-segregating with the *w* allele had a negative effect. Effects of specific haplotypes were similar if they originated from the S or the LW population, however, their effects were smaller if they originated from the LR population (paired T-test, *P* < 0.05). For each breed the average effects of the *m* and *w* allele, weighted according to the haplotype frequencies, are shown as red numbers in Fig. 4. The difference of the averages is an approximation of the allele substitution effect, substituting an *m* allele for a *w* allele has an effect on ADG of − 2.5 g/d, − 0.5 g/d and − 1.6 g/d, when the allele originates from S, LR, or LW, respectively. Using the MC4Rsnp itself, the effect of substituting an *m* allele for a *w* allele at MC4Rsnp was − 22.60 g/d, − 14.21 g/d, or − 21.67 g/d, when the allele originated from S, LR or LW, respectively. Figure 5 shows the number of times each haplotype was observed per breed-of-origin versus its effect on crossbred performance for ADG. For S breed-of-origin, there is one very common haplotype accounting for 73% of the observations and this haplotype had the largest effect (+ 1.52 g/d) among all the haplotypes in this LD block. For LR breed-of-origin, the 19 haplotypes observed had small effects, from − 0.40 to + 0.54 g/d, and the most common haplotype accounted for 37% of the observations and had an effect of − 0.11 g/d. For LW breed-of-origin, the haplotypes had more variable estimated effects, and the most common haplotype accounted for 28% of the observations and had an effect of − 1.16 g/d.

## Discussion

The objective of this study was to show how the effect of SNP-alleles, estimated in a genomic prediction model using commonly used SNP panels, varies when observed in different genetic backgrounds. With crossbreeding, the effects of SNP-alleles can be observed both against purebred and crossbred background. Moreover, the degree of allelic differentiation among the three populations estimated with Weir and Cockerham’s *F*_*ST*_ was previously estimated by Sevillano et al. [18] and were equal to 0.17 between S and LR, 0.12 between S and LW, and 0.14 between LW and LR, which indicates that they are distantly-related breeds. Since the three breeds are distantly-related, the effects of the SNP-alleles is expected to vary in the three distinctive backgrounds provided by each of the breeds-of-origin.

To observe the estimated effects of SNP-alleles for crossbred performance in different genetic backgrounds, we traced the breed-of-origin of alleles in crossbred animals and estimated breed-specific SNP effects from the solutions of a BOA model for three traits. For traits with low heritability (< 0.20) and low r_pc_ (< 0.70) the BOA model tended to show better predictive abilities [18]. Therefore, based on the heritability and r_pc_ estimates with pedigree information from Godinho et al. [19], BF, ADG and RFI, were chosen to be studied. Only for RFI, the estimated heritability for crossbred performance differed from the expected value of ~ 0.2 [19] as it was considerably higher (0.40) in our data. Genetic parameters estimated for LR pigs had high standard errors because of the limited number of RFI records, therefore, GEBVs of LR pigs for purebred performance were not further used in this study. For all the other traits, estimates of r_pc_ and heritability for crossbred performance were as expected.

### Proportion of genetic variance explained by a region

The proportion of total genetic variance explained was calculated for each LD block instead of reporting effects of single SNPs. The LD blocks built based on alleles originating from the S paternal population were, on average, longer than the LD blocks built based on alleles originating from the maternal LR and LW populations. This is in line with linkage disequilibrium estimations made by Veroneze et al. [42] using the same populations as in our study, where they showed that the S population showed the highest level of linkage disequilibrium, followed by LW, and LR having the lowest level of linkage disequilibrium. Their populations named SL2, DL1 and DL2 correspond to S, LR and LW populations in the present study.

#### Regions associated with purebred and crossbred performance

Within the same breed-of-origin, we observed some LD blocks that appeared for both purebred and crossbred performance in the top 10 with most explained genetic variance. Across traits, this number of common LD blocks in the top 10 is expected to be related to the r_pc_ for that trait, as the correlation between allele substitution effects of the causal variants of two traits is expected to be the same as the genetic correlation between two traits [43]. Our results are in line with this, as RFI showed the lowest r_pc_ (0.37–0.60) and had at most only one LD block that appeared for both purebred and crossbred performance, while BF showed the highest r_pc_ (0.71–0.89) and had 4 to 5 LD blocks that appeared for both purebred and crossbred performance.

For LD blocks that appeared for both purebred and crossbred performance in the top 10 with most explained variance, we observed that they explained a similar percentage of additive genetic variance. Despite the fact that percentages of additive genetic variance were quite similar, differences in allele frequencies between purebred and crossbred can explain r_pc_ values below unity. However, as shown in Table 8, allele frequency of the MC4Rsnp between purebred and crossbred were quite similar. One of the possible reasons for r_pc_ values below unity is the presence of genotype by environment interactions (GxE) [19, 43]. GxE might have been present because some purebred pigs were housed in high-health status farms (nucleus farms, free of a number of specific diseases), while some crossbred pigs were housed in experimental farms with environmental conditions similar to commercial farms with these specific diseases prevalent. Another environmental difference between purebred and crossbred is that trait measurement methods were different [19, 43], as explained earlier in the methods section. ADG and BF were measured in a different way for purebred and crossbred pigs, and as these are the components traits for RFI, RFI was also derived differently for purebred and crossbred. It is unclear to which extent the genetic ranking is affected by these differences in measurements. Nevertheless, using crossbred information in the training set avoids that the difference in measurements affects the breeding decisions.

#### Regions associated with crossbred performance by breed

Next to the comparison between purebred and crossbred background, comparison across breed-of-origin backgrounds was also performed. For all traits, there was at most one region in common between breeds-of-origin for crossbred performance. This indicates that the proportion of genetic variance for crossbred performance explained by a genomic region depends upon the breed-of-origin. Differences in genetic variance across breeds-of-origin can be due to differences in allele frequencies that affect the contribution of dominance effects to the additive genetic variance. The allele frequency of the MC4Rsnp (see Table 8) is quite different in crossbred pigs depending upon the breed-of-origin. In addition, for the block co-segregating with the MC4R gene originated from the LR population, we observed a relatively small variance among the effect sizes of the different haplotypes, caused by the low frequency of the *m* allele of MC4Rsnp (Fig. 5). For S and LW, we observed that the haplotypes in this region had a larger variance of effect size for ADG performance in crossbred, because the MAF of the MC4Rsnp was considerably higher. We hypothesize that for other genomic regions, similar differences in MAF may be one important source of differences in how the genetic variance is distributed across the genome for different breeds, and therefore having different contribution to genomic prediction.

We also observed that the effect of a haplotype associated with crossbred performance is different depending upon from which population it originates. In the case of MC4R, identical haplotypes co-segregating uniquely either with the mutant or the wild type allele, yielded different effects for LR compared to S and LW (Fig. 4). Similarly, the effect difference between haplotypes co-segregating with the *m* allele and the *w* allele was five and three times larger for haplotypes originating from S and LW compared to haplotypes originating LR, respectively (Fig. 4). Differences in haplotype effects across breed-of-origin can be due to differences in linkage between the haplotype and any QTL in the vicinity, however, this was not the case for MC4R. Another reason for these differences in haplotypes effects across breed-of-origin might be that the haplotypes are not identical between the breeds, they only appear to be so due to the genotype resolution used. If that is the case, the difference can be due to distinct interactions of the MC4R allele with different local genetic background, i.e., epistasis [9]; or because the unobserved differences between the haplotypes directly give rise to additional additive effects. So, what is observed as a breed-of-origin effect may actually be different haplotypes which can be only differentiated with a higher density genotype. However, when we estimated the allele substitution effect of the MC4Rsnp itself, we still observed that the largest effect was when the allele originated from S, followed by LW origin, while alleles from LR origin had the smallest effect. But the magnitude was much larger than when we approximate the allele substitution effect from the haplotypes estimates. These differences might arise from the methodology as SNP effects in the haplotypes were estimated jointly as random effects via BLUP, being subjected to considerable shrinkage, whereas MC4Rsnp effects were estimated using fixed regression. For the MC4Rsnp we can conclude that the main difference across breeds are the allele frequencies which can reflect selection pressure for other performance traits, as also observed by Kim et al. [30].

In general we observed few regions strongly associated with ADG, RFI, or BF for crossbred performance, and these are mainly breed-specific. Conversely, we observed many regions that did not have a large effect on ADG, RFI, or BF for crossbred performance. Hypothesizing that only for regions with large effect breed-specific modelling is beneficial, using SNP effects averaged across breeds may be more realistic than considering breed-specific SNP effects. We previously compared the BOA model, which considers breed-specific SNP effects in crossbred animals, to a model that does not account for breed-specific SNP effects in crossbred animals [18], and found similar or slightly higher accuracies of estimated breeding values with the BOA model. This suggests that few regions, such as the region containing the MC4R, may benefit from accounting for breed-specific SNP effects.

## Conclusions

Some similar regions explaining similar additive genetic variance were observed across purebred and crossbred performance. The number of similar regions was related to the trait r_pc_. Observed r_pc_ values below one can be due to differences in housing and trait measurements between purebred and crossbred as they can affect the genetic ranking. Therefore crossbred information is valuable in the training set to account for the environmental background differences between crossbred and purebred performance.

Moreover, there was some overlap across breeds-of-origin between regions that explained relatively large proportions of genetic variance for crossbred performance of ADG, RFI, and BF; albeit that the actual proportion of variance deviated across breeds-of-origin. This variation is due to differences in allele frequencies across population and epistasis can be also playing a role. Results based on a missense mutation in MC4R confirmed that even if a causal locus has similar effects across breeds-of-origin, estimated effects and explained variance in its region estimated using a genomic prediction model relying on a SNP panel can strongly depend on the allele frequency of the underlying causal mutation.

These results are valuable to understand the limited benefit obtained when predicting breeding values of purebred animals for crossbred performance with models that account for breed-specific effect of alleles, as the BOA model, compared to a model using crossbred information but without accounting for breed-specific effect of alleles. However, selecting important regions associated with crossbred performance and differentiating their SNP-allele effects according to their breed-of-origin, might improve prediction models for crossbred performance.

## Additional files

Additional file 1: Proportion of genetic variance for back fat thickness explained by the top 10 LD blocks for purebred and crossbred performance by breed-of-origin. (PDF 214 kb)

Additional file 2: Proportion of genetic variance for average daily gain explained by the top 10 LD blocks for purebred and crossbred performance by breed-of-origin. (PDF 214 kb)

Additional file 3: Proportion of genetic variance for residual feed intake explained by the top 10 LD blocks for purebred and crossbred performance by breed-of-origin. (PDF 215 kb)
