# Supplementary material for: Effects of alleles in crossbred pigs estimated for genomic prediction depend on their breed-of-origin
Source: BMC Genomics. 2018 Oct 11;19:740. doi: 10.1186/s12864-018-5126-7 (PMC6180412; doi:10.1186/s12864-018-5126-7)
Supplement: Supplementary file 1 — Proportion of genetic variance for back fat thickness explained by the top 10 LD blocks for purebred and crossbred performance by breed-of-origin. (PDF 214 kb) [file 12864_2018_5126_MOESM1_ESM.pdf]

S								LR								LW							
**Chromosome**	**# snp**	**Start position, bp**	**End position, bp**	**rank PB**	**rank CB**	**gVar PB, %**	**gVar CB, %**	**Chromosome**	**# snp**	**Start position, bp**	**End position, bp**	**rank PB**	**rank CB**	**gVar PB, %**	**gVar CB, %**	**Chromosome**	**# snp**	**Start position, bp**	**End position, bp**	**rank PB**	**rank CB**	**gVar PB, %**	**gVar CB, %**
1	17	158940596	160210902	10	9	0.24	0.31	1	27	53883921	55119115	20	6	0.12	0.19	1	13	12517775	12779800	60	9	0.10	0.23
4	21	2020990	2923924	45	8	0.11	0.33	2	28	10790595	11683915	7	13	0.15	0.14	1	16	159238083	160210902	5	28	0.26	0.14
5	7	29291278	29807656	6	13	0.31	0.24	4	9	17843084	18032705	5	>	0.17	0.03	2	16	9821331	10521615	7	1	0.25	0.38
5	16	30079766	30783586	4	6	0.35	0.39	4	4	76050292	76120254	>	9	0.04	0.17	2	6	144583667	144699882	6	4	0.25	0.29
6	20	45162579	46159717	13	7	0.21	0.37	5	5	66721316	66841468	8	>	0.15	0.05	2	7	144841166	145072905	1	6	0.35	0.26
6	27	47770178	49883373	5	3	0.32	0.53	6	12	4410006	4812421	27	5	0.11	0.22	5	3	66211719	66307363	4	36	0.27	0.12
11	10	7556280	7940249	16	5	0.20	0.40	6	26	5671575	6181785	23	8	0.11	0.17	5	11	91894653	92261586	21	7	0.16	0.26
11	15	7959313	8,759,687	14	4	0.20	0.47	6	19	66750992	67959109	10	31	0.15	0.10	6	24	77495951	78368681	2	2	0.31	0.37
11	22	8940153	9859528	11	10	0.22	0.30	6	15	85959532	86401903	6	19	0.16	0.13	9	24	87025203	91501337	10	23	0.21	0.15
14	4	111029177	111497364	9	>	0.24	0.02	8	26	60556630	63983448	3	3	0.18	0.31	10	4	25784747	25911585	9	30	0.22	0.13
15	30	102308093	104508529	2	2	0.64	0.59	9	20	67988	611480	4	2	0.17	0.32	11	10	7556280	7940249	3	3	0.29	0.29
15	25	124052987	124718596	7	>	0.29	0.05	9	17	127384290	127871583	9	24	0.15	0.12	11	27	7959313	9394153	23	5	0.15	0.29
17	5	19434891	19639253	3	>	0.48	0.01	9	24	128346581	128912053	1	1	0.25	0.33	15	21	118193036	118772018	34	10	0.12	0.22
18	13	9589537	10036306	8	31	0.27	0.14	14	13	14247493	14909953	32	7	0.10	0.18	15	17	132454779	132854143	17	8	0.17	0.26

18	8	10083200	10555467	1	1	1.62	1.14	15	21	119257860	119820905	2	4	0.21	0.30	16	17	32932796	33391365	8	29	0.22	0.14
								16	6	35614962	35917453	35	10	0.10	0.15								
Total[1]						4.51	4.51	Total[1]						1.73	2.35	Total[1]						2.61	2.85

[1]Total measured only considering the top 10 blocks

> Ranking higher than 100.

gVar PB = percentage of genetic variance explained by a LD block for purebred performance.

gVar CB = percentage of genetic variance explained by a LD block for crossbred performance.
