# Supplementary material for: Effects of alleles in crossbred pigs estimated for genomic prediction depend on their breed-of-origin
Source: BMC Genomics. 2018 Oct 11;19:740. doi: 10.1186/s12864-018-5126-7 (PMC6180412; doi:10.1186/s12864-018-5126-7)
Supplement: Supplementary file 2 — Proportion of genetic variance for average daily gain explained by the top 10 LD blocks for purebred and crossbred performance by breed-of-origin. (PDF 214 kb) [file 12864_2018_5126_MOESM2_ESM.pdf]

S								LR								LW							
**Chromosome**	**# snp**	**Start position, bp**	**End position, bp**	**rank PB**	**rank CB**	**gVar PB, %**	**gVar CB, %**	**Chr**	**# snp**	**Start position, bp**	**End position, bp**	**rank PB**	**rank CB**	**gVar PB, %**	**gVar CB, %**	**Chr**	**# snp**	**Start position, bp**	**End position, bp**	**rank PB**	**rank CB**	**gVar PB, %**	**gVar CB, %**
1	22	22995675	23914711	8	22	0.19	0.13	1	30	24160760	25558722	9	2	0.16	0.21	1	37	148170231	150386584	2	1	0.44	0.52
1	17	50797783	51394966	1	2	0.67	0.71	2	9	127150044	127601163	4	53	0.18	0.08	1	6	152357354	153127120	4	8	0.32	0.21
1	32	51502130	52696117	2	1	0.62	0.87	3	25	10101710	11076493	74	10	0.07	0.14	1	16	154108314	155462235	1	2	0.63	0.37
1	13	52764397	53673007	65	10	0.10	0.19	3	10	95567908	95998761	10	>	0.15	0.05	1	16	159238083	160210902	3	34	0.33	0.13
1	11	53753531	54125627	4	4	0.46	0.34	4	34	123951734	125150793	2	50	0.23	0.08	1	13	160903291	162372950	9	7	0.21	0.22
1	17	158940596	160210902	60	5	0.10	0.33	8	9	128638260	128759315	8	>	0.16	0.04	3	9	89799364	90371246	45	6	0.11	0.22
6	14	27029375	28268828	34	9	0.12	0.20	9	13	21695319	22071228	71	8	0.08	0.15	5	10	21487563	21970939	10	>	0.18	0.07
9	46	76052645	78754343	7	6	0.20	0.25	9	16	85529908	86702813	7	>	0.16	0.06	7	34	27394424	28541774	24	9	0.14	0.21
12	2	2269903	2291228	6	>	0.21	0.03	10	4	41980415	42035062	34	1	0.10	0.22	7	16	37145252	37994461	21	5	0.14	0.22
12	50	2371834	4006859	87	3	0.08	0.48	10	12	59598915	60001020	19	9	0.12	0.15	8	7	11342418	11417428	8	22	0.21	0.14
15	25	124052987	124718596	5	>	0.23	0.01	10	19	60023220	60408029	69	3	0.08	0.18	10	8	36258440	36584543	7	99	0.23	0.07
15	20	132308830	132854143	10	>	0.18	0.02	12	28	16536280	17289226	1	4	0.37	0.17	15	12	8009455	8319700	32	10	0.12	0.19
16	36	41412816	44459651	18	8	0.16	0.20	13	28	28111755	29212714	>	6	0.03	0.16	15	17	132454779	132854143	6	3	0.23	0.30
17	11	11211527	11438330	46	7	0.11	0.22	15	15	78438274	79022296	6	16	0.18	0.13	18	20	53234165	53887250	5	4	0.23	0.27

17	1	19407489	19407489	9	>	0.19	0.00	18	6	9757116	9935903	3	5	0.20	0.17								
17	5	19434891	19639253	3	>	0.62	0.00	18	11	44962762	45188783	5	>	0.18	0.05								
								18	12	54261734	54696517	15	7	0.13	0.15								
Total[1]						3.57	3.80	Total[1]						1.81	1.71	Total[1]						3.00	2.71

[1]Total measured only considering the top 10 blocks

> Ranking higher than 100.

gVar PB = percentage of genetic variance explained by a LD block for purebred performance.

gVar CB = percentage of genetic variance explained by a LD block for crossbred performance.
