# Supplementary material for: Effects of alleles in crossbred pigs estimated for genomic prediction depend on their breed-of-origin
Source: BMC Genomics. 2018 Oct 11;19:740. doi: 10.1186/s12864-018-5126-7 (PMC6180412; doi:10.1186/s12864-018-5126-7)
Supplement: Supplementary file 3 — Proportion of genetic variance for residual feed intake explained by the top 10 LD blocks for purebred and crossbred performance by breed-of-origin. (PDF 215 kb) [file 12864_2018_5126_MOESM3_ESM.pdf]

S								LR								LW							
**Chromosome**	**# snp**	**Start position, bp**	**End position, bp**	**rank PB**	**rank CB**	**gVar PB, %**	**gVar CB, %**	**Chr**	**# snp**	**Start position, bp**	**End position, bp**	**rank PB**	**rank CB**	**gVar PB, %**	**gVar CB, %**	**Chr**	**# snp**	**Start position, bp**	**End position, bp**	**rank PB**	**rank CB**	**gVar PB, %**	**gVar CB, %**
2	13	8862251	9150134	10	>	0.20	0.05	1	13	183924206	185042994		1		0.53	1	24	10362059	10911742	7	>	0.19	0.06
2	25	133590610	134496051	>	6	0.01	0.21	6	46	14539308	16224309		3		0.26	1	30	24499758	25948260	9	>	0.19	0.10
2	33	134538298	135282067	>	1	0.15	0.42	6	10	21728055	21946695		4		0.24	1	37	148170231	150386584	70	8	0.09	0.19
3	15	111556261	111940811	>	10	0.00	0.18	6	12	23955670	24925892		5		0.24	1	13	183719835	184802857	3	2	0.25	0.29
4	21	2020990	2923924	3	4	0.29	0.25	6	56	62235659	65483397		2		0.29	1	14	246897187	247304332	>	4	0.04	0.25
4	19	94185015	94967342	>	5	0.01	0.23	12	28	16536280	17289226		6		0.19	3	20	98977271	100368914	2	36	0.26	0.12
5	13	5149762	5344082	>	3	0.00	0.28	14	47	45827810	47826895		7		0.19	4	8	104606569	104793040	5	>	0.23	0.06
5	16	78361124	79093904	62	9	0.09	0.20	16	24	18490090	19483752		8		0.17	4	18	105565718	106658684	24	3	0.13	0.26
6	11	155346307	155602352	7	>	0.22	0.04	17	5	29056018	29246809		9		0.16	6	31	14945308	16264536	1	68	0.38	0.09
7	33	2203524	2849221	>	2	0.05	0.30	18	6	47607716	47709763		10		0.16	6	16	107231177	108437309	21	1	0.14	0.32
10	10	1810290	2029001	2	39	0.37	0.11									7	20	2582366	2954492	>	5	0.02	0.20
10	18	3253139	3720144	8	>	0.21	0.04									7	16	37145252	37994461	10	60	0.19	0.10
12	45	8846410	9767814	1	36	0.59	0.12									10	9	46083236	46267311	8	41	0.19	0.11
14	47	46219359	48033173	>	8	0.06	0.21									12	21	9792506	10162364	6	>	0.21	0.03

14	23	125343446	125929866	9	7	0.21	0.21									12	23	16484131	17116574	29	6	0.13	0.20
15	14	14560948	14903909	5	>	0.23	0.07									13	16	52757976	53401107	>	10	0.02	0.18
16	33	18133808	19357238	4	12	0.25	0.17									15	42	27445103	28658318	4	51	0.24	0.11
18	21	47239897	47709763	6	40	0.23	0.11									15	26	127657851	128198698	33	9	0.12	0.18
2	13	8862251	9150134	10	>	0.20	0.05									17	14	46801041	47554481	>	7	0.02	0.19
2	25	133590610	134496051	>	6	0.01	0.21																
Total[1]						2.80	2.50	Total[1]							2.42	Total[1]						2.33	2.28

[1]Total measured only considering the top 10 blocks

> Ranking higher than 100.

gVar PB = percentage of genetic variance explained by a LD block for purebred performance.

gVar CB = percentage of genetic variance explained by a LD block for crossbred performance.
